# Temporal trend evaluation in monitoring programs with high spatial resolution and low temporal resolution using geographically weighted regression models

**DOI:** 10.1007/s10661-023-11172-2

**Published:** 2023-04-10

**Authors:** Claudia von Brömssen, Jens Fölster, Karin Eklöf

**Affiliations:** 1 Division of Applied Statistics and Mathematics, Department of Energy and Technology, Swedish University of Agricultural Sciences, PO Box 7032, 750 07 Uppsala, Sweden; 2 Section for Geochemistry and Hydrology, Department of Aquatic Sciences and Assessment, Swedish University of Agricultural Sciences, PO Box 7050, 750 07 Uppsala, Sweden

**Keywords:** Geographically weighted regression, Temporal trend assessment, Brownification, Surface waters, Low temporal monitoring frequency

## Abstract

Data from monitoring programs with high spatial resolution but low temporal resolution are often overlooked when assessing temporal trends, as the data structure does not permit the use of established trend analysis methods. However, the data include uniquely detailed information about geographically differentiated temporal trends driven by large-scale influences, such as climate or airborne deposition. In this study, we used geographically weighted regression models, extended with a temporal component, to evaluate linear and nonlinear trends in environmental monitoring data. To improve the results, we tested approaches for station-wise pre-processing of data and for validation of the resulting models. To illustrate the method, we used data on changes in total organic carbon (TOC) obtained in a monitoring program of around 4800 Swedish lakes observed once every 6 years between 2008 and 2021. On applying the methods developed here, we identified nonlinear changes in TOC from consistent negative trends over most of Sweden around 2010 to positive trends during later years in parts of the country.

**Supplementary Information:**

The online version contains supplementary material available at 10.1007/s10661-023-11172-2.

## Introduction

Surveillance monitoring has two main aims: (i) to assess the status of a target object and (ii) to detect long-term trends over time (Carvalho et al., 2019; Fölster et al., 2014b). Typically, for the work to be cost-effective, different types of monitoring programs with varying temporal and spatial resolution are established. A few selected stations are monitored frequently (at least once a year) to evaluate and quantify trends, while general status is assessed at a larger number of stations, but with lower temporal frequency (e.g., once or twice in a 6-year-period; Carvalho et al., 2019). The data from the latter are often disregarded in trend evaluations or only used to provide auxiliary information. In this study, we examined whether geographically weighted regression models can reveal the spatially detailed and unique information provided by monitoring programs with high spatial resolution and low temporal resolution and if this information can be utilized to quantify regional trends, i.e., trends driven by large-scale phenomena such as atmospheric deposition or climate.

Rotating panel, also called serially alternating, designs consist of a large number of monitoring sites that are split into a few panels, which are revisited at regular intervals, but with differing start years. Some panels may be revisited annually, in what is called an augmented rotating design (McDonald, 2003). Several approaches based on linear mixed models for analyzing trends in the data obtained in such programs have been suggested (Starcevich et al., 2018a, b; Urquhart & Kincaid, 1999). For example, Dauwalter et al. (2010) estimated trends individually for each site and, using these, computed a mean slope across a larger area. Starcevich et al. (2018a) used a mixed model comprising a fixed joint trend slope, random site-specific trend slopes, random effects for sites and years, and a random site-year interaction (Piepho & Ogutu, 2002) for this purpose. These methods are designed to evaluate a single population-level trend, under the assumption that this trend has the same form and similar magnitude over the entire geographical region under assessment, while site-specific trends also can be evaluated using the random-effect site-level trend slopes. This assumption is reasonable for smaller areas or for regions where a single large-scale driver affects all sites uniformly. However, in many cases, it is naïve and could conceal important information. Moreover, due to the low temporal resolution of the data, conventional station-wise trend detection methods, such as Mann–Kendall tests (Hirsch & Slack, 1984; Hirsch et al., 1982), are not applicable, as they require more than a few data points per series. There is thus a lack of appropriate statistical methodology for evaluating temporal trends that vary over space in data with sparse temporal resolution.

Spatially varying relationships without a temporal component can be studied using geographically weighted regression (GWR) models (Brunsdon et al., 1998a). Such models allow the relationship between two variables to change smoothly over space and have been applied widely in social, economic, and medical studies. In environmental studies, Tu and Xia (2008) and Taghipour Javi et al. (2014) used GWR models to connect water quality or quantity to land use indicators, while Koh et al. (2020) studied nutrient contamination in groundwater in relation to hydrological and land use variables. In many applications of GWR, the analysis is based on single observations per site, object, or administrative unit. To counterbalance that, the regression model for a specific object uses a geographical window. Observed objects within this window, adjusted by weights that represent the distance to the object of interest, are used to build the regression model. GWR has also been adapted to include additional spatial correlations (GWR-SAR; Brunsdon et al., 1998b) and both spatial and temporal weights (GTWR; Fotheringham et al., 2015; Huang et al., 2010). For space–time data also geographically weighted panel regression models have been suggested (Yu, 2010). While the latter methods make use of temporal data, the focus was on the spatial representation and not changes in time. Since GWR is a computationally expensive method, several solutions for parallel computations have been suggested and implemented (Harris et al., 2010a, b; Murakami et al., 2021; Wang et al., 2020).

Geographically weighted regression models are a useful tool for exploring geographically diverse temporal trends in temporally sparse data as long as these trends are influenced by large-scale drivers, i.e., can be assumed to be spatially homogeneous to some extent. In this study, we examined whether GWR models can be modified by adding time as an explanatory variable and adjusting the approach used for pre-processing of data. This would permit the evaluation of spatially differentiated trends and thereby uncover new information about patterns of prevailing trends. Specifically, in this study, we sought to determine whether:

1. Pre-processing of data could be performed to lower the influence of outliers and remove small-scale variation in observed levels of the variable of interest.
2. The size of the chosen k-nearest-neighborhood (knn) influences observed large-scale geographical patterns and which size works best for the problem at hand.
3. Datasets with higher temporal resolution can be added to increase the available information in sparse datasets, in order to produce more reliable temporal trends.
4. Nonlinear temporal trends can be identified using an approach with temporally moving windows.

For illustration purposes, we used data from the Swedish Lake Survey (SLS), a monitoring program covering around 4800 lakes with a revisit interval of 6 years, that has not been utilized previously to study temporal trends. Brownification of surface waters was selected as an example of an issue that would benefit from the development of relevant statistical tools to evaluate spatial–temporal data. Brownification may be driven by recovery from acidification (Evans et al., 2005; Monteith et al., 2007) and by changes in climate, such as increased temperature or precipitation (de Wit et al., 2016; Weyhenmeyer & Karlsson, 2009; Weyhenmeyer et al., 2012). However, there are also alternative theories on the causes of brownification with more local effects, including, e.g., changes in land use and increasing coniferous forest cover (Kritzberg, 2017; Meyer-Jacob et al., 2019; Škerlep et al., 2020). An ability to compute regionally differentiated trends for brownification variables on a dense spatial grid would provide a clearer picture of which of these hypotheses is most important. As the focus of this study was on the statistical methodology, we used only one variable, total organic carbon (TOC). A more extensive analysis to evaluate potential drivers of brownification with the proposed statistical methods was conducted in a parallel study and will be reported elsewhere.

## Methods

### Geographically weighted regression models

Geographically weighted regression models are used to model a response variable as a function of one or several explanatory variables ${x}_{1}, {x}_{2}, \dots , {x}_{p}$ (Brunsdon et al., 1998a; Comber et al., 2023):

$${Y}_{i}={\beta }_{i0}+\sum_{k=1}^{p}{\beta }_{ik}{x}_{ik}+{\varepsilon }_{i}$$

where ${\beta }_{ik}$ are the regression coefficients for the variable ${x}_{ik}$, *k* denotes the different variables, and *i* the spatial location. This formula is valid for each of the n locations and contains usually no temporal component. The error term in the model is assumed to follow a normal distribution with a mean zero.

This means that for each spatial location, a regression model is defined. In order to achieve spatial smoothing of the regression coefficients, the coefficient for a specific location is computed from observations within a spatial neighborhood. Such a neighborhood can be defined using a fixed distance band or as a group of stations formed by the *k* nearest neighbors. Within the neighborhood, weights are assigned to individual observations, depending on the distance of the observations to the location modeled. Then, a weighted least squares regression is computed using the regression equation and the defined weight matrix. A typical choice for the weights makes use of the Bisquare weighting function (Brunsdon et al., 1998a):

$$w_{ij}=\left\{\begin{array}{c}\left(1-\left(d_{ij}/b\right)^2\right)^2\;\;\;if\left|d_{ij}<b\right|\\\;\;\;\;\;\;\;\;\;0\;\;\;\;\;\;\;\;\;\;\;\;\;otherwise\end{array}\right.$$

where ${d}_{ij}$ denotes the distance between observations *i* and *j*, while *b* represents the physical distance from location *i* to the *k*^*th*^ nearest neighbor. Standard errors for the estimated GWR coefficients correspond to standard errors in a weighted least squares regression (Fotheringham et al., 2002; Harris et al., 2010a, b).

To make this method available for use in trend analysis, we reformulated the model with respect to two features: (i) we allowed several observations instead of single observations per location and (ii) we used time explicitly as an explanatory variable. While it is possible to add additional explanatory variables, we concentrated on the basic trend model. The model is thus:

$${Y}_{ij}={\beta }_{i0}+{\beta }_{i}{t}_{ij}+{\varepsilon }_{ij}$$

where *i* again denotes the geographical location and *j* the *j*^th^ observation at that location, and ${t}_{ij}$ is a time variable, typically a year of observation.

Including time as an explanatory variable requires adjusted pre-processing of the data (see section “Data pre-processing”). We set the size of the neighborhood to be adaptive, as the spatial sampling density varied and used a Bi-square kernel to spatially weight the observations. We created the GWR model using the package GWmodel (Gollini et al., 2015; Lu et al., 2014) in the free statistical software R (R Core Team, 2022). As the dataset was rather large and GWR models are computationally expensive, we opted to perform computations in parallel on a standard PC using the default option provided in GWmodel. The size of the k-nearest-neighborhood (knn) was determined by cross-validation and also compared to user-defined sizes of the neighborhood. R scripts and data used in this study are available online (von Brömssen et al., 2023).

### Data

The Swedish Lake Survey has monitored approximately 4800 lakes since 2007 (Fölster et al., 2014a, b). This program has a revisit time of 6 years, and each year a panel consisting of about 800 lakes is monitored, leading to time series of two or three observations per location so far. The spatial monitoring density is higher in southern Sweden. Mid-lake surface samples are taken during autumn circulation, usually between September and December, since these measurements are regarded as representative of the entire lake (Göransson et al., 2004). The selection of lakes is made randomly within geographical strata and accounting for lake size. Only lakes larger than 0.01 km^2^ are included (Grandin, 2007). Occasional late observations (up to January) are attributed to the autumn of the previous year. During autumn 2018 (including January 2019), only 463 lakes were sampled due to a technical breakdown of the helicopter used for monitoring. The remaining lakes were sampled together with the panel planned for 2019, leading to a total of 1080 lake measurements in 2019. Some data quality problems were detected for the first year of monitoring in the SLS (2007), so our analysis was based on data from 2008 to 2021. As the goal was to identify larger regions with common trends, the 11 lakes on the island of Gotland, which lies off the south-east coast of Sweden, were omitted from the analysis.

A parallel monitoring program, the Swedish Trend Lake (STL) program, initially covered small acidification-sensitive forest lakes, which were not treated by liming to mitigate acidification. Over time, the scope was broadened several times, and it now includes lakes that are unaffected by local point source pollution in general. Its monitoring sites have a good geographical representation in Sweden, but with a higher density in southern Sweden. The current program includes 107 lakes (excluding lakes on the island of Gotland). Most of the lakes are monitored four times a year, while some have a more frequent sampling regime (Fölster et al., 2014a). For the purposes of the present study, where STL data were co-analyzed with SLS data, STL surface water observations (≤ 1 m) during autumn, i.e., between September and December, in the years 2008 to 2021 were selected. When evaluating nonlinear trends, all available STL data between 2008 and 2021 were used for a supporting analysis (section “Nonlinear temporal trends”).

All data used were downloaded from the environmental data internet service at the Swedish University of Agricultural Sciences https://miljodata.slu.se/MVM/.

### Data pre-processing

#### Basic pre-processing

The goal of our study is to conduct temporal trend evaluation and we removed, thus, sites with less than two observations to ensure that only time series type of data were included. This led to the removal of 214 observations/stations. All TOC data were log_10_-transformed before analysis to account for the skewed data distribution and to decrease the effect of potential outliers.

#### Detection of series with high variation and outliers

Local outliers can strongly influence GWR estimates (Comber et al., 2023; de Bellefon & Floch, 2018; Fotheringham et al., 2002). A number of adjusted methods have been suggested to reduce the potential influences of single observations, by identifying and eliminating or downweighting them (Fotheringham et al., 2002; Harris et al., 2010a, b), by using robust estimation (Sugasawa & Murakami, 2021; Zhang & Mei, 2011), or by robust cross-validation (Farber & Páez, 2007; Harris et al., 2010a, b). In this study, the log_10_-transformation of the data before analysis already decreased the effect of single deviating observations. Additionally, we took advantage of the fact that we had several observations per site, which is usually not the case for GWR. We were thus able to identify sites with unusually large variation relatively to observed levels by computing the coefficient of variation (CV) for each station as defined for log-normal data (Koopmans et al., 1964):

$${CV}_{log-normal}=\sqrt{{e}^{{s}_{ln}^{2}}-1}$$

where ${s}_{ln}^{2}$ is the variance in the response on the scale of the natural logarithm. Here, we used a logarithm with base 10, so this was substituted with ${s}_{ln}={s}_{10}\bullet ln\left(10\right)$, where ${s}_{10}$ is the standard deviation of the response transformed with a logarithm of base 10. Station-wise variances and station-wise mean values were also computed and compared.

#### Assessing the geographical heterogeneity of temporal changes

To evaluate whether a geographically differentiated trend model was necessary, we first fitted a global model, i.e., we assumed a homogeneous trend slope for all of Sweden and fitted a traditional linear regression on time while ignoring spatial dependence. For this model, we studied the geographical heterogeneity of the residuals using plots and summary statistics. To further assess the spatial autocorrelation of a feature, Moran’s I, which is a measure for clustering of spatial data, can be computed (Comber et al., 2023). Here, we wanted to determine whether the predominant trend differed in different parts of Sweden and thus we determined the spatial clustering using the change over time, rather than the level of the variable of interest. For this, we determined the change per year for each individual station as:

$${change}_{year}=\frac{{y}_{j}-{y}_{i}}{j-i}$$

where ${y}_{i}$ is the first log-transformed observation at a site and ${y}_{j}$ is the last observation, while *i* and *j* indicate the years of observation. We then computed Moran’s I for the variable *change*_*year*_ using Euclidian distances in space, with the package ape in R (Paradis & Schliep, 2019).

#### Station-wise centering of data

**Fig. 1:**
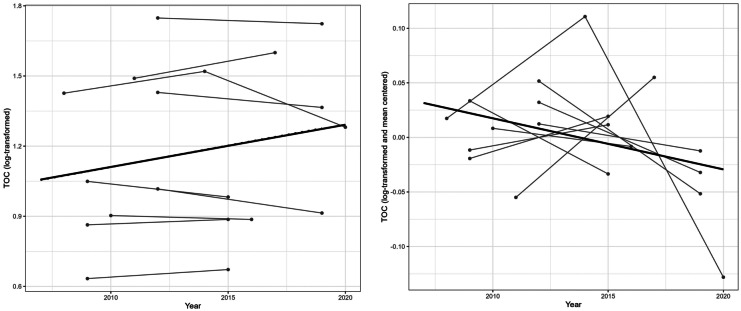
(Left) Log-transformed values and a joint regression line for data from nine stations in a geographical window in Sweden and (right) data from the same nine stations, log-transformed, and mean-centered with a joint regression line

When data are collected from a number of sites, the general level of the variable of interest can vary due to factors such as catchment land uses and the depth, type, or size of the lake. While we assumed that the prevailing temporal trends in a neighborhood are similar, we could not make this assumption for the levels of the variable of interest. Since GWR is based on the spatial weighting of observations and the data analyzed here were collected with a low temporal resolution, varying station-wise levels might lead to undesired effects on the trend analysis. To illustrate this, observations of log-transformed TOC from nine stations within a geographical window are shown in Fig. 1 (left panel). Both decreasing and increasing trends are evident at individual stations, but several of the stations with higher mean levels were monitored during later years. A joint trend estimated for the data (here without geographical weighting) led to the conclusion that there is an apparent upward trend, with an estimated magnitude of 0.018 units per year (standard error: 0.02). To decrease the effect of station-specific mean levels, we applied station-wise mean-centering of the data, i.e., we subtracted the mean level of each station from the individual observations, resulting in a series with zero mean (Fig. 1, right). When we then computed the trend slope for these series, the result was a slightly negative trend estimate (−0.005; standard error: 0.002).

### Model validation

For a GWR model, the residual sum of the square, local R^2^, and *p*-values can be computed to assess its fit (Comber et al., 2023). The *p*-values can be corrected for multiple testing using, e.g., Benjamini and Hochberg False Discovery rates or the Bonferroni method (da Silva & Fotheringham, 2016). In our application, *p*-values were only used to identify areas with potential trends and exact *p*-values were not relevant. Therefore, we followed the recommendation of Brunsdon et al. (1998a) and only considered areas with a large proportion of locally very distinct trends, while smaller areas and single stations indicating geographically limited deviations were ignored. Stations that were identified in the pre-processing step as containing much variation compared with the overall level of the measurements were examined by computing the differences between the estimated trend slopes obtained for models with and without these stations. While we generally used the size of the geographical neighborhood suggested by the cross-validation approach, we also compared these results with a model assuming a much smaller neighborhood, both visually and by computing the difference between the estimated trend slopes for the two neighborhood sizes.

### Combining datasets

Geographically weighted regression involves the weighting of observations from geographical windows, which makes it possible to combine data from different monitoring programs even if their temporal and spatial resolution vary. Here, we merged SLS and STL data, with SLS being a random sample of lakes and STL a sample of lakes unaffected by local pollution sources. It was thus important to verify that the same type of overall trend was monitored, especially since the STL monitors with substantially higher temporal frequency than the SLS. The effect of including the STL data in the analysis was evaluated by inspecting the changes obtained in slope estimates.

### Nonlinear temporal trends

GWR allowed us to estimate linear trends in time, but for longer time series, this would not be adequate to identify ongoing changes. Therefore, in addition to the overall GWR analysis, we also applied GWR on temporal subsets, so-called moving windows, of the SLS data. We used coefficients estimated for 10-year-long windows and results plotted side-by-side to identify any clear changes in the trend slope coefficients over time, i.e., any indications of nonlinearity.

For comparison, we evaluated nonlinear trends in the STL data, which have a higher temporal resolution, using generalized additive mixed models (Hastie & Tibshirani, 1986; Wood, 2017) and the visualization methods suggested by von Brömssen et al. (2021). For this analysis, we used all available data in the STL, usually at least one observation per season. We modeled the overall trend by a thin plate spline and accounted for seasonal variation in the model by a seasonal indicator variable with four levels: spring (March–May), summer (June–August), autumn (September–November), and winter (December–February). The error term is modeled as an autoregressive process of order 1 to account for the temporal autocorrelation in these time series.

## Results

### Data quality assurance and pre-processing

**Fig. 2:**
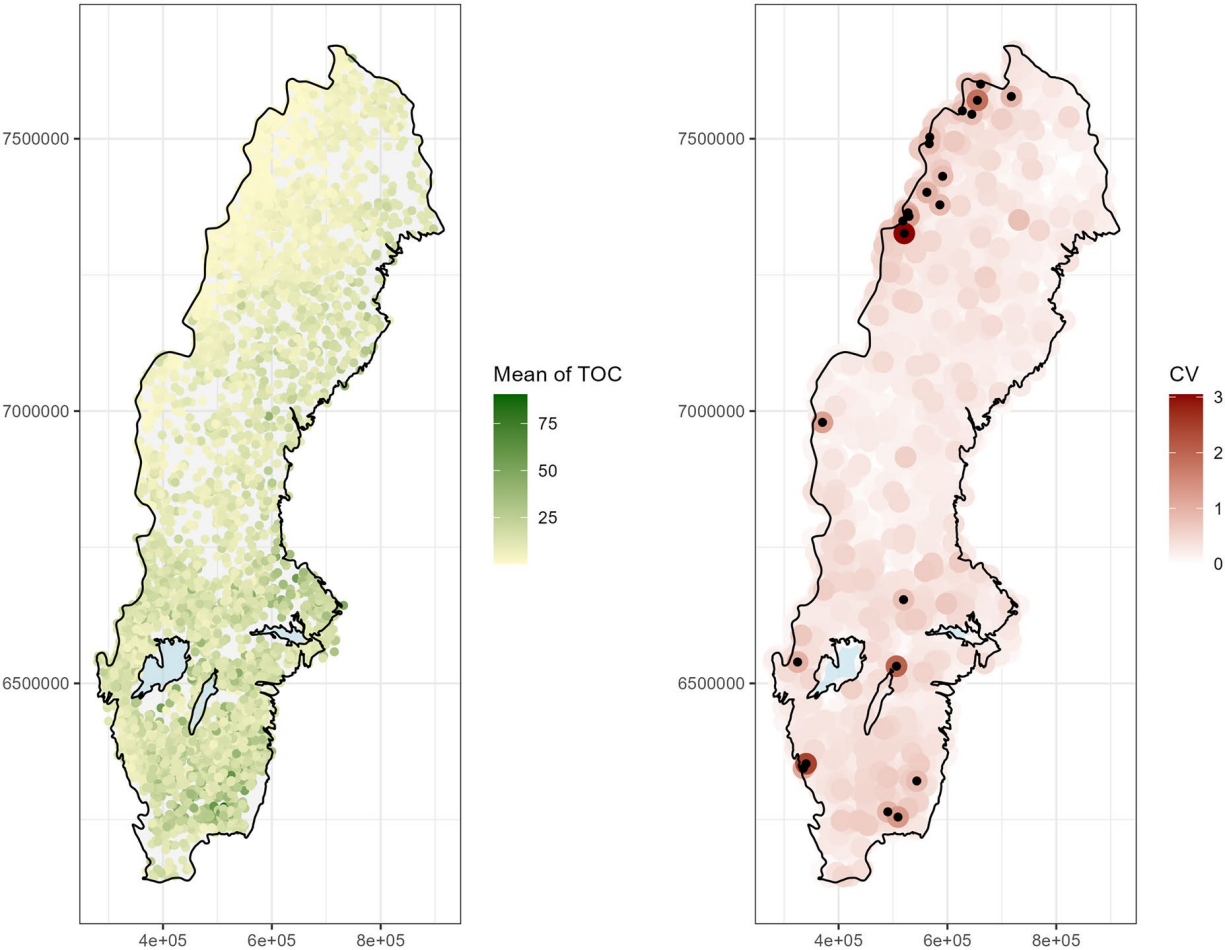
(Left) Mean levels of total organic carbon (TOC, mg/L) observed in lake water at monitoring stations throughout Sweden and (right) coefficient of variation (CV_log-normal_) for observed values. Black dots indicate stations with a coefficient of variation larger than 0.8

High levels of TOC were generally recorded in southern Sweden, but also on the north-east coast (Fig. 2, left). Using the coefficient of variation, we were able to locate stations with relatively high variation compared with the station mean (Fig. 2, right). Especially, eleven lakes had a value higher than 1 (Table S1 in Supporting Information (SI)), i.e., the standard deviation of the log-transformed observations was at least as large as their local mean. An unusually high number of such lakes (five) were observed in the Swedish alpine region (north-west of the country), where the overall level of TOC in lake water is generally low (≤ 1 mg/L). At four of these five stations, unusually high TOC levels were observed as the first recorded value, which could introduce an artificial trend if these observations were faulty. However, the measurements were made during different years, and there is no obvious reason to believe that these observations were not correct. The remaining stations with the highest coefficient of variation were distributed randomly over the country and included stations that so far only have been visited twice. While these stations most probably have some effect on trend estimation in their immediate region, at this stage we were unable to determine whether these were invalid or erroneous measurements and they needed to be revisited instead in model validation. Station-wise variances were computed, but did not contribute further to the analysis of data quality.

### Trends in TOC

Moran’s I was calculated on the change per year based on individual stations and showed a spatial pattern (*p* < 0.001). This pattern was confirmed by fitting a global linear trend model and analyzing its residuals (not shown). Thus, both analyses indicated a need for geographically differentiated analysis.

**Fig. 3:**
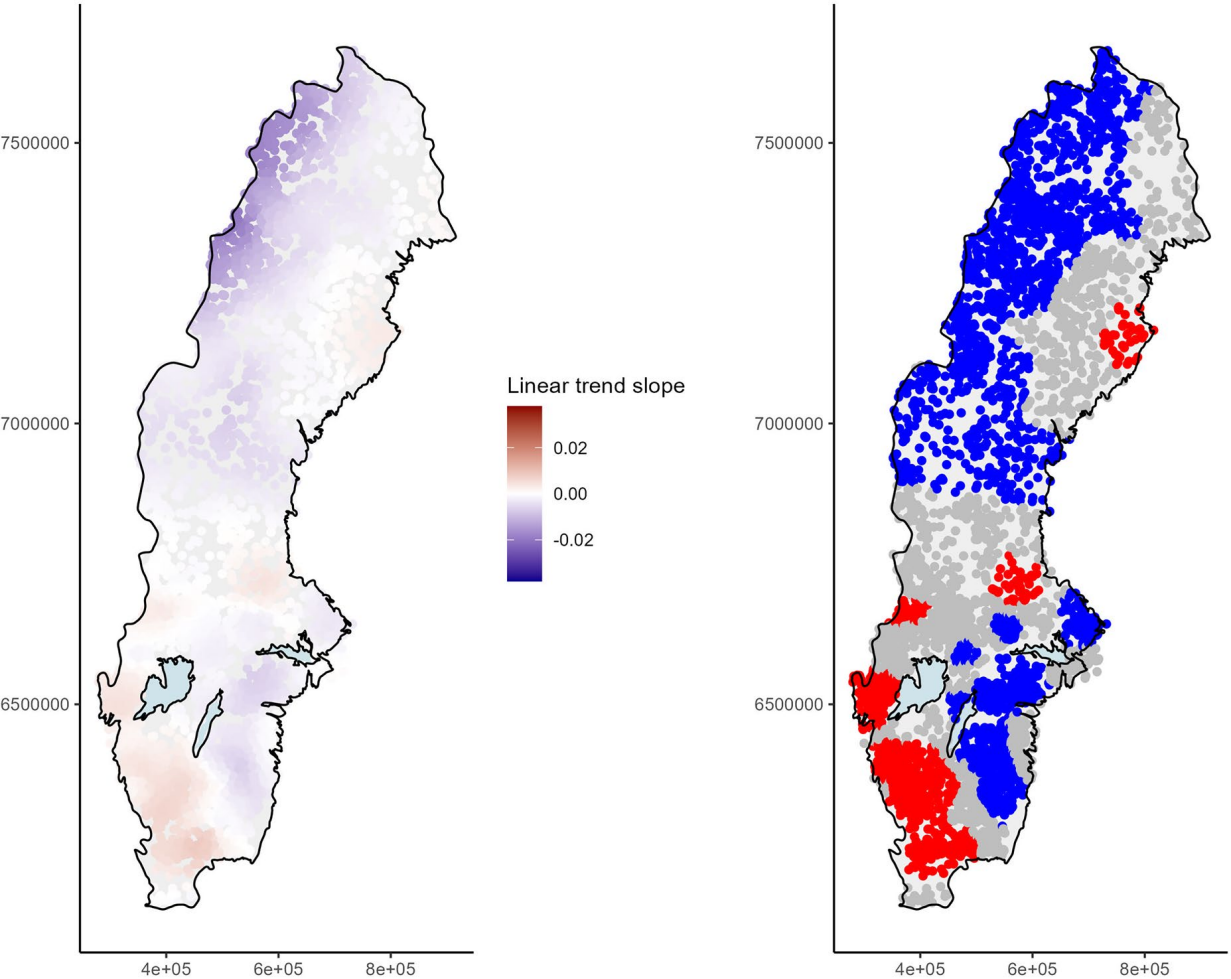
(Left) Estimated linear trend slopes of log-transformed and mean-centered total organic carbon (TOC) concentrations in Swedish lake water from 2008 to 2021 and (right) distribution of slopes significant at 5% level in geographically weighted regression analysis based on k-nearest-neighborhood (knn) of 370 observations, where blue indicates a significant downward trend, red significant upward trend, and gray no significant trend. *P*-values were not adjusted to multiple testing

Temporal trends in TOC were estimated by GWR for log-transformed TOC values that were mean-centered within the station. The size of the k-nearest neighborhood was determined to be 370 by cross-validation, indicating that approximately 125 lakes (about 2.5% of all lakes), with varying weights, were considered in the estimation of the trend slope for an individual lake. Clear decreasing trends with the magnitude of about 0.02 were observed in the Swedish alpine region (Fig. 3, left) and trends with slightly lower magnitude in south-east Sweden. Increasing trends were seen in south-west Sweden and to some extent in the region of Gävleborg in central Sweden. A plot illustrating where trends were significant at an unadjusted significance level of *p* < 0.05 was used to enhance this geographical information (Fig. 3, right).

For comparison, GWR was also computed for data without station-wise mean-centering, where the cross-validated k-nearest neighborhood was determined to be 23, i.e., only about 8 lakes. While the overall picture of positive and negative trend slope estimates in some regions still could be identified (Fig. S2, left) local deviations were much larger due to the small size of the selected neighborhood and standard errors of trend slopes were approximately 10 times higher than in the centered case (Figs. S1 and S2, right). Only few stations in the alpine region showed significant trends. Similarly, using a larger neighborhood (knn = 370) few regions showed significant trends due to the increased local variation when the TOC level of stations differed strongly and the standard errors of the trend slopes were still about 4 times larger compared to the model for mean-centered data. The model using non-centered data was not considered further.

### Model validation

The resulting model had an overall adjusted *R*^2^ value of 0.12, with local *R*^2^ values up to about 0.4 in the region with the strongest trends, i.e., the alpine region. The standard error values computed in GWR showed the expected pattern of higher variation in regions with the sparser geographical resolution, especially along the north-east coast, central-west Sweden, and southernmost Sweden (Fig. S1 in SI). Regions with high trend slopes and significant trends were mostly well connected. Some smaller areas with significant trends were observed, especially in central Sweden (Fig. 3, right), but these trends should not be interpreted unless supported by additional data, expert knowledge, or auxiliary information from relevant background variables. The removal of 24 lakes with CV_log-normal_ > 0.8 obviously led to a change in trend estimates, but the change was seldom larger than 0.001 (Fig. S3 in SI, left). This was mainly observed in the alpine region, where the negative trends appeared weaker on removing high-variation sites. Due to the lower variation in some other regions, new stations appeared to be significant after the removal of several high-variation sites. However, these were randomly scattered throughout Sweden and trends were thus not eligible for interpretation.

**Fig. 4:**
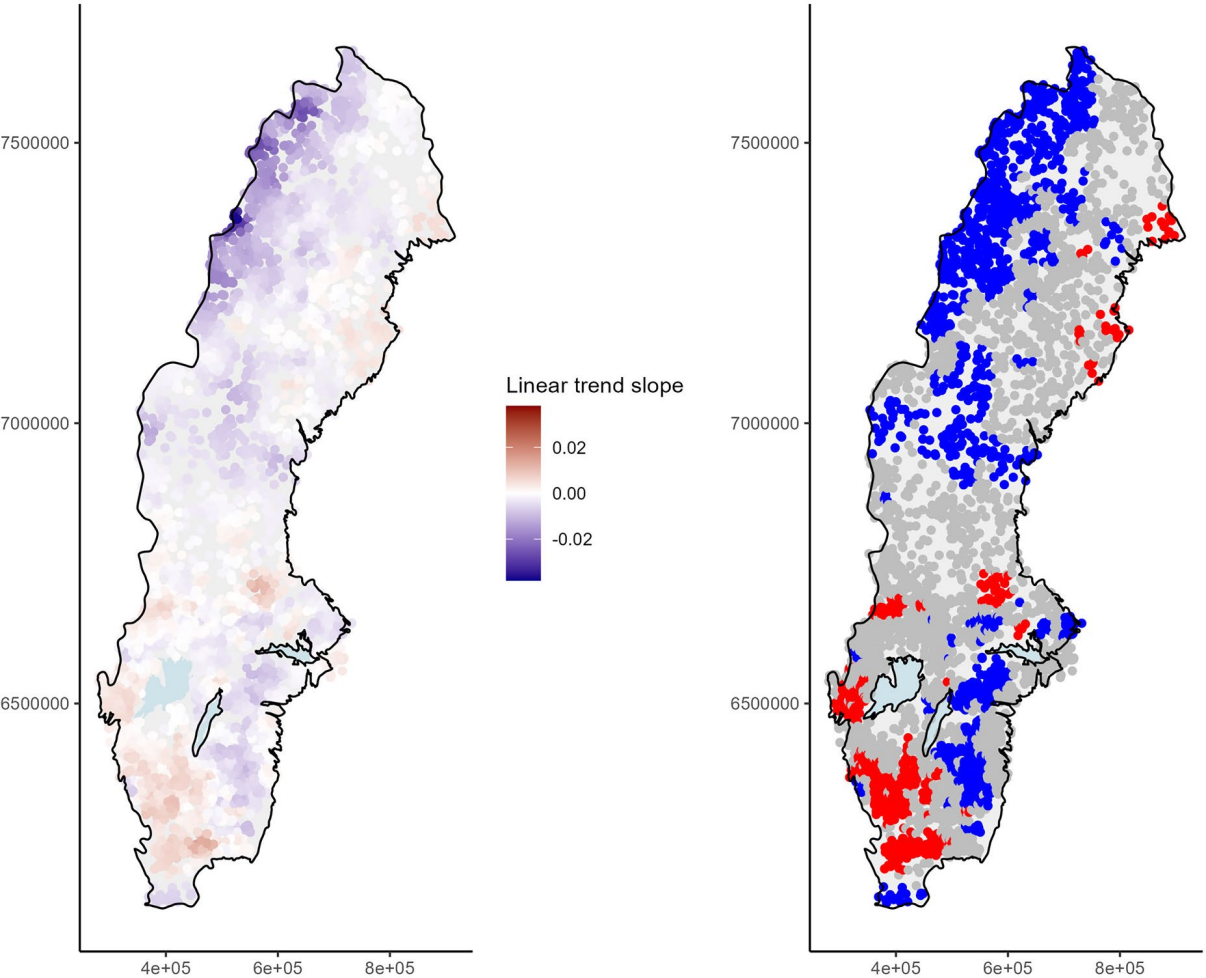
(Left) Estimated linear trend slopes of log-transformed and mean-centered total organic carbon (TOC) concentrations from 2008 to 2021 and (right) distribution of slopes significant at 5% level in geographically weighted regression analysis based on k-nearest-neighborhood (knn) of 90 observations, where blue indicates a significant downward trend, red significant upward trend, and gray no significant trend. *P*-values were not adjusted to multiple testing

It was expected that single lakes would not have any substantial influence on the results of this model, as the neighborhood used was rather large. However, the large neighborhood also made it more difficult to observe local trends that could arise due to climatic or atmospheric deposition effects on a smaller scale (e.g., rainy alpine regions, high deposition on south-west coast) or to detect whether smaller regional drivers had more effect than large-scale drivers. We evaluated the effect of neighborhood by running a GWR with a smaller k-nearest-neighborhood of only 90 observations (approx. 30 lakes). The overall picture was unchanged, with decreasing trends in the alpine region, central-west Sweden, and south-east Sweden and increasing trends mainly in the south-west (Fig. 4, left). However, local trends were much more pronounced, with maximum slope estimates as high as 0.037 (for negative trends in the alpine region; standard error: 0.002) and 0.015 (for positive trends around Hanöbukten; standard error: 0.002) and local *R*^2^ values above 0.7. Consequently, fewer locations showed significant trends (Fig. 4, right), and significant trends were more often observed for small clusters of lakes, especially around the edges of the fitted area, i.e., the coastline. Even for the smaller neighborhood (knn = 90), the removal of lakes with high CV_log-normal_ did not change the overall picture (results not shown).

### Combining data from different monitoring programs

**Fig. 5:**
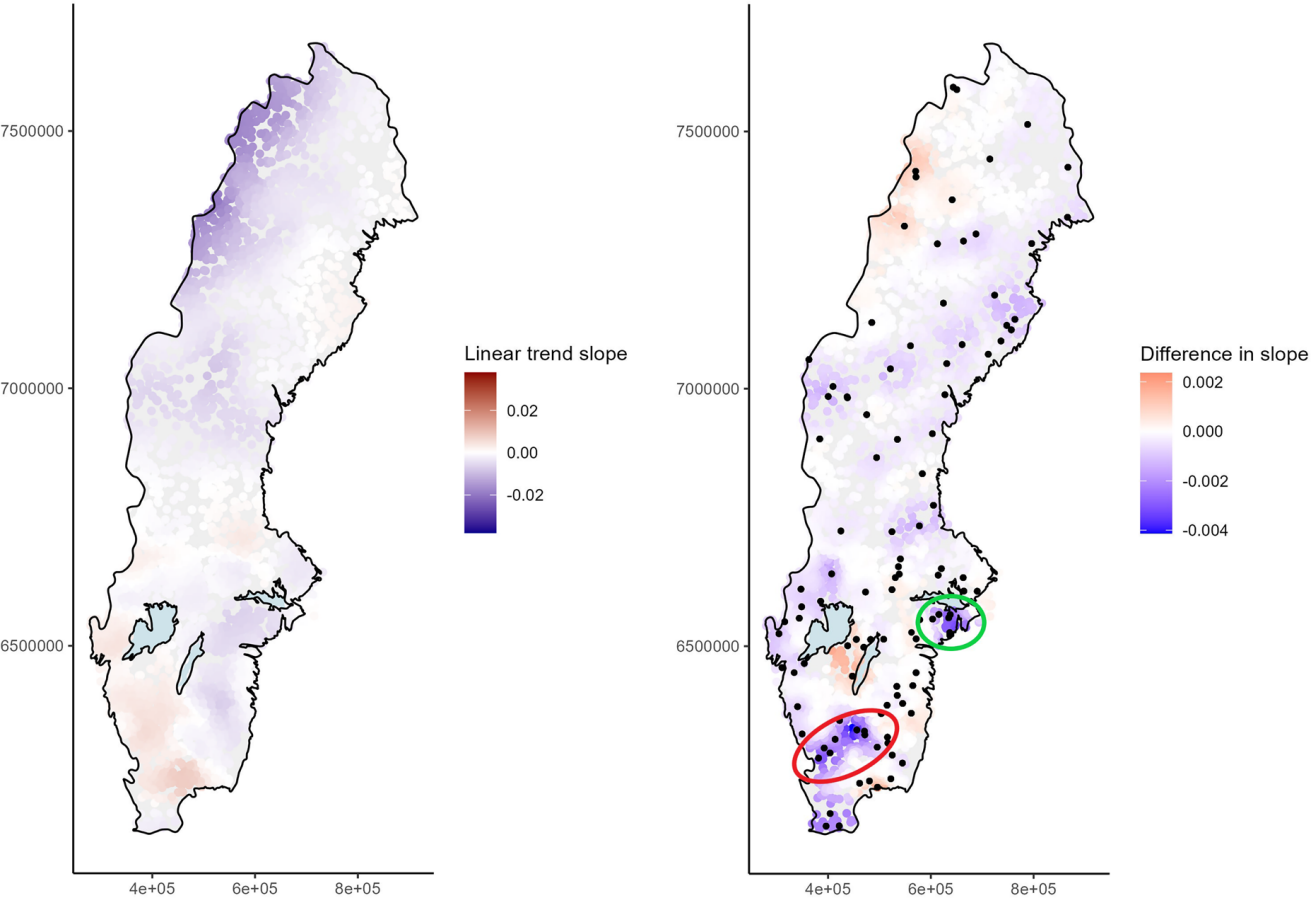
(Left) Estimated linear trend slopes of log-transformed and mean-centered total organic carbon (TOC) concentrations from 2008 to 2021 when both SLS and STL data was used and (right) differences computed as the estimated coefficient in the combined model minus the coefficient when only SLS data were used. Black points in the right panel indicate the location of STL monitoring sites, while circles indicate two region with pronounced changes after the inclusion of STL data

**Fig. 6:**
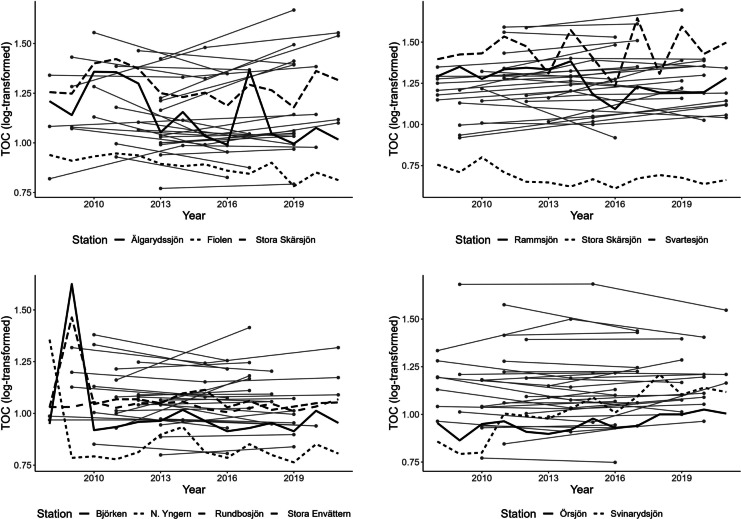
Changes in total organic carbon (TOC) concentrations over time in lakes within a geographical window of 30 Swedish lakes around (upper left) Lake Kupesjön near Värnamo, (upper right) Älvasjön near Halmstad, (lower left) around Norasjön near Södertälje, and (lower right) outside Karlshamn. The data series for Swedish Lake Survey (SLS) lakes are shown in gray, and the data series for Swedish Trend Lakes (STL) is in black

The STL program has a higher temporal frequency, but much fewer stations than the SLS program. We merged data from these two programs and conducted a joint GWR. Cross-validation determined the optimal k-nearest neighborhood to be 400 for the combined data (about 130 lakes or 3% of all stations). While the overall picture of prevailing trends was quite similar to that observed for the SLS data alone (Fig. 5, left), there were some differences in the estimated trend coefficients (Figs. 5, right; S3, right). The greatest difference in the estimated slope coefficient was observed around Lake Hindsen in the extreme south of Sweden, but since this is an area with few lakes on the edge of the observed geographical area, we did not interpret this change further. A similar difference in slope estimate was observed around Lake Kupesjön, which lies near three STL lakes, Älgarydssjön, Fiolen, and Stora Skärsjön. All three STL lakes showed some decrease in TOC concentration over the years (Fig. 6, upper left), while SLS lakes mainly indicated increased TOC level or no change. This area connects with lakes north-east of Halmstad (Fig. 6, upper right), where a similar change was observed (Fig. 5 (right), red circle). In both areas, the overall estimated trend magnitude was still mainly positive, but was lessened by the addition of the STL lakes. The approximate decrease in trend slope estimate was 0.0035 units. A third area with decreased slope coefficient after the inclusion of the STL lakes was identified south of Stockholm, around Södertälje. There, most of the STL lakes exhibited high values during the first years in the series (Fig. 5 (right), green circle; Fig. 6, lower left), a finding which needs to be studied further. Clear confirmation of an increasing trend in TOC was given by the STL lakes Svinarydsjön and Örsjön outside Karlshamn, close to Hanöbukten (Fig. 6, lower right). The estimated trend slope for this region was around 0.002 units steeper when STL lakes were included compared with only SLS lakes.

### Nonlinear trends in TOC

**Fig. 7:**
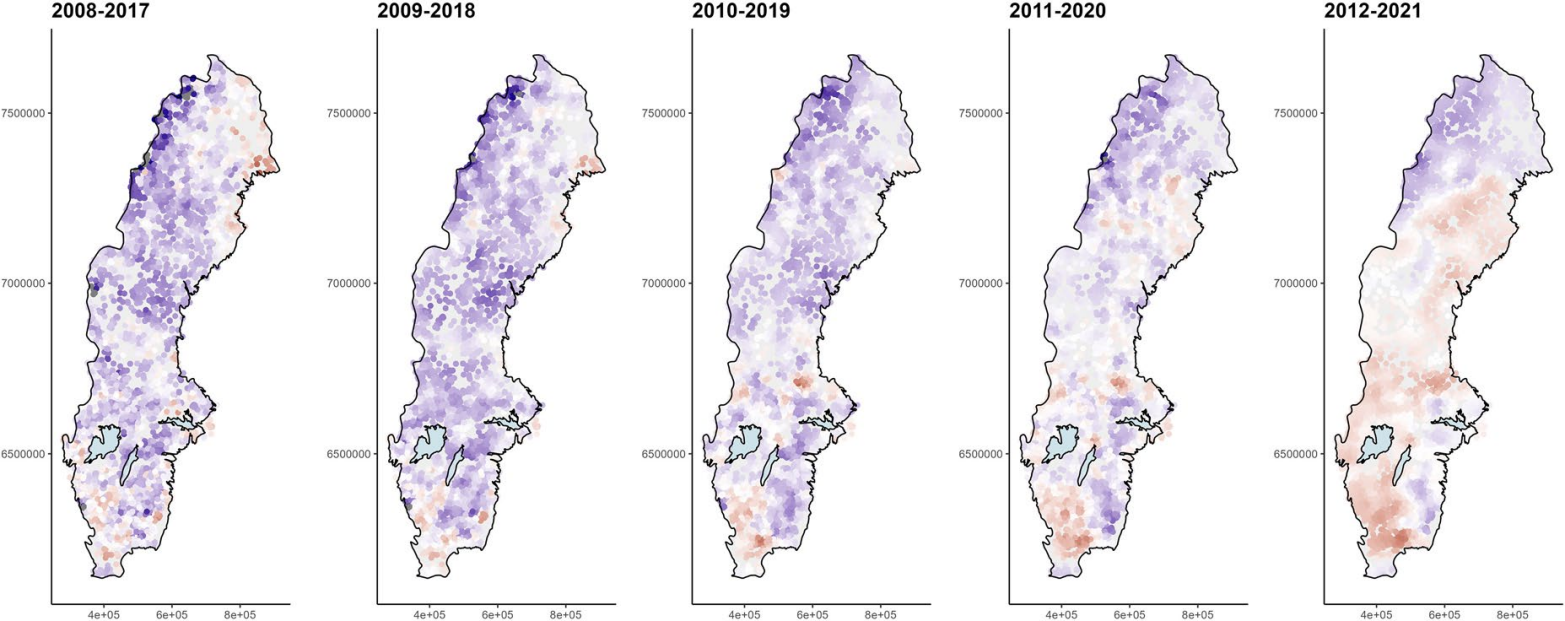
Estimated linear trend slopes of log-transformed and mean-centered total organic carbon (TOC) concentrations in Swedish lake water for five 10-year-long temporal windows based on k-nearest-neighborhood (knn) of 29, 51, 63, 61, and 45 observations, respectively. Blue indicates negative trend coefficients, red positive trend coefficients, and white trend coefficients close to zero

To study nonlinear trends in the SLS data, we fitted separate GWR models in five different 10-year temporal windows (2008–2017, 2009–2018, 2010–2019, 2011–2020, 2012–2021). We observed primarily downward trends in TOC during the first few 10-year periods (Fig. 7, left), while, especially during the last 10 years, we observed increasing TOC levels in south-west and north-east Sweden (Fig. 7, right). Negative trends prevailed in the northern Swedish alpine regions and, to a lesser extent, in the south-east during the entire observation period. Even though the periods used in the analysis were only 10 years long, many of the observed changes identified were significant (Fig. S4 in SI).

To validate the results, we again used the sites in the STL program. As the data had a higher temporal resolution, we performed the analysis of nonlinear trends station-wise for the 107 stations, using generalized additive models. We observed that for TOC, significant downward trends were present even in this analysis for several lakes in the north, as well as the south east (e.g., Glimmingen, Fjärasjö, and Hökesjön), and several series exhibited upward trends towards the end of the series, both in north-east (e.g., Täftesträsket, Hällvattnet) and in south Sweden (Fig. S5 in SI). In the south, the most prominent increases happened in Västergötland, between the large lakes Vänern and Vättern, while no or even negative trends were observed in the south-east, as already observed in the combined SLS and STL analysis. An exception was again the area around Hanöbukten where lakes Svinarydsjön and Örsjön showed significant increases.

## Discussion

### GWR as a method for trend evaluation for datasets with a sparse temporal resolution

Large amounts of data are collected in environmental monitoring to determine the status of lakes, water courses, grasslands, forests, or other types of objects. A typical choice for such programs is to keep the temporal resolution of the series low in favor of obtaining a better spatial representation and including more objects in the survey. Still, such extensive data sets are also very interesting for temporal trend analysis. GWR has so far not been used to evaluate temporal trends but is a much-used tool to quantify spatially varying relationships. Including time as an explanatory variable in GWR, we can conduct a trend analysis as long as trends can be assumed to be consistent over a reasonably large region. In the brownification of surface waters, climate and current and previous acid deposition are expected to influence the level and change in TOC (de Wit et al., 2016; Evans et al., 2005; Monteith et al., 2007; Weyhenmeyer & Karlsson, 2009; Weyhenmeyer et al., 2012), and Sweden has a clear gradient both in deposition and climate. Therefore, TOC in Swedish lakes is a suitable study area for geographically differentiated temporal trends as identified by GWR.

Even though GWR is quite straightforward to use for temporal trend evaluation by including time of observation as an explanatory variable, there are some additional features to be considered. For example, the level of observed quantities is typically not influenced by a single driver, but by a combination of characteristics of the site in question. This is most probably also the cause for TOC, as trends of TOC have been suggested to be driven by land use (Škerlep et al., 2020) and soil processes related to recovery from acidification (Evans et al., 2012). Such local differences in confounding factors can easily disguise existing trends or create artificial trends, especially if the geographical window chosen is small. To avoid such effects, we used two approaches: (i) station-wise mean-centering of data as a pre-processing step to rescale data at a mean of zero and thus remove site observed levels as an influencing factor; and (ii) evaluation of the chosen window size. We used adaptive kernels defined as k-nearest neighbors (knn), as the data had a varying spatial resolution, which makes a fixed bandwidth problematic (Comber et al., 2023). Cross-validation is recommended to choose the best local size of the knn. In our case, the cross-validation results suggested a neighborhood of approximately 372 observations or 125 lakes. Smaller neighborhoods allow more local trends to manifest, which can be of interest if the spatial resolution is sparser, i.e., if a large neighborhood oversmooths results or if drivers are expected to work on a smaller regional scale. With very sparse temporal data, as in the present case, it will generally not be possible to identify trends on a small geographical scale. Instead, the goal must be the rough identification of areas with increasing and decreasing TOC levels, while the exact borders of these regions are not important. In this study, analyses with larger and smaller knn produced very similar results, with clear decreases in TOC in the northern alpine region and in south-east Sweden and with increasing TOC levels primarily in south-west Sweden.

It needs to be pointed out that for our approach GWR is a hypothesis-generating rather than a hypothesis-testing method. Provided results include standard errors for trend estimates as well as *p*-values, but both of these will be underestimated due to several reasons. First, temporal autocorrelation might be present in addition to the temporal trend, but is not possible to estimate in time series that only consist of two or three observations. Similarly, spatial correlation might still be present in the error term even after geographical smoothing. The presence of either of these correlations will lead to underestimation of standard errors, which in turn will cause too low p-values. Additionally, as we have a large number of locations, adjustment for multiple testing (da Silva & Fotheringham, 2016) would be needed to decrease the risk of false positives. To avoid these issues, we chose to only interpret patterns of trends and with that lay the foundation to generate hypotheses on where trends are present and, consequently, which drivers might be of importance.

### Pre-processing and model validation

As GWR models are sensitive to outliers (Brunsdon et al., 1998a), pre-processing and model validation are important. We did this by identifying stations with a high coefficient of variation before model fitting and analyzing the effect of the removal of such stations on the model results. A high coefficient of variation may mean stations that have faulty high or low values, but can also indicate stations that have a steep temporal trend. In the latter case, their removal would obviously be disadvantageous for trend evaluation. Since the removal of such stations with a high coefficient of variation had little effect in the present analysis, this indicates that they were stations with a steep trend located in areas that generally showed such a trend, or single stations, which would not be interpreted as trends anyway. For small knn in particular, we found several limited areas with significant trends along the Swedish coastline and in the alpine region. This is not unexpected, as it is generally more difficult to estimate reliable trend coefficients at the edge of the observable area (Leong & Yue, 2017), and single stations with a distinct increase or decrease can have larger effects.

The overall and local *R*^2^ values can be used to determine the goodness of fit. For our main model, the overall *R*^2^ was only 0.12. However, this was not informative as there were large regions in Sweden that did not exhibit a trend and therefore contributed to a low coefficient of determination. The local *R*^2^ values are more interesting to quantify, and those reached 0.4 for the model with a large neighborhood size and 0.7 when a small neighborhood was chosen, indicating that there are rather strong temporal trends in some geographical areas. Consequently, the GWR approach is deemed successful as it can identify regions with trends as well as such without. The inclusion of local covariates might improve the overall and local *R*^2^ values additionally and will be studied separately. Care needs, however, to be taken as high R^2^ values in models with very small neighborhoods or with many covariates might be a sign of overfitting due to the small number of observations available per station.

Additionally, GWR models can be validated by comparing results with studies with a similar objective but using datasets with higher temporal resolution. For example, Eklöf et al. (2021) studied TOC concentrations and absorbance in 164 watercourses distributed over Sweden and found a smaller number of streams with ongoing increasing TOC trends in the period 2011–2020, after a period of more general positive trends. Most of these streams were located in south-west Sweden and the along the north-east coast, in agreement with the findings in our study. Eklöf et al. (2021) also identified single stations in the south-east with decreasing trends in TOC during the past decade, but in that region and in the alpine region only a few water courses were sampled and results are more difficult to compare.

To validate the results of a trend analysis, it could be interesting to conduct a power study. Pregler et al. (2019) conducted a power study for different revisit designs and found that serially alternating designs have similar power to always revisited designs if the total amount of observations is the same. Similarly, Starcevich et al. (2018a) found that a revisit design with more unique sites is preferable to annual revisit panels of sites when detecting trends over at least 12 years of monitoring with a linear mixed model. However, both assume that a common trend is present over the area of interest. In our case, it is reasonable to assume that trends differ in different parts of Sweden and the power to detect spatially differentiated trends would thus be more difficult to quantify.

### Combining datasets with a low and high temporal resolution

By using a separate dataset, the STL data, we were able to add data from 107 additional stations in computing the GWR model. The data for the STL stations have a higher temporal resolution, but do not represent randomly selected stations, which the SLS does. Instead, the STL program monitors stations that are mainly unaffected by local pollution sources. As the main goal in the present case was to study trends caused by large-scale drivers, such as climate, and recovery from acid deposition, the different objectives of the two surveys were not expected to affect the trend evaluation results to any substantial degree. Rather, the STL data helped make a distinction between large-scale drivers that were of interest here and more local pollution sources. We compared the results obtained in combined analysis with those based on the SLS alone and found only a few differences. The most noticeable change occurred in south-west Sweden, between Halmstad (approx. 56.692748 lat., 13.112771 long.) and east of Värnamo (approx. 57.208242 lat., 14.142123 long.), where the estimated trend slope for TOC decreased by 0.0035 units compared to the SLS dataset alone, meaning that local pollution sources might be prevalent for lakes that are part of the SLS. However, the combined analysis results still indicated an increase in TOC levels. For comparison, in the well-studied Lake Bolmen water color has been observed to decrease during the past decade (Klante et al., 2021), following high levels observed after a strong storm and large amounts of tree felling in 2005. Such nonlinear temporal relationships could also be a reason why different monitoring programs with different objectives provide information that is not completely in line.

In other regions of Sweden, the STL data supported or enforced the general direction of the SLS trends. For example, near Karlshamn, the STL lakes showed clear increasing trends, which was also observed in the SLS lakes. Similarly, the downward trend in the Helags alpine region was confirmed by data from the STL program. This is an area where results based on the SLS alone must be considered uncertain, due to large variations and low absolute values of TOC and its situation at the edge of the observable space.

### Nonlinear temporal trends

In the present study, we assumed that the temporal trend in log-transformed TOC can be approximated by a line, but is allowed to vary smoothly over space. Linear temporal trends can be reasonable if time series are short or, as in our case, data are sparse and linearity is a helpful simplification. However, to understand trends and what is driving these, it is necessary to investigate in nonlinearity whenever possible. We addressed this using a moving window approach, i.e., we subdivided the data into five 10-year intervals, moving the interval 1 year forward in each step. This provided at least some information about how estimated trend slopes changed over time. In particular, we found that during the last 10-year period studied (2012–2021), lakes increasingly showed positive trends after a period of an overall decrease in TOC. The reliability of the results of this nonlinear analysis was confirmed on comparing them to the results from trend analysis based on higher frequency data. Here, we used a screening of trends with generalized additive models (von Brömssen et al., 2021), which showed increases in TOC at a number of stations during the latest years of monitoring.

The possible presence of nonlinear trends in data puts our initial analysis into question. A combination of data with sparse temporal resolution and nonlinear trends will require some trade-off in modeling. Here, the goal was to demonstrate the usefulness of GWR in such situations, and we present both the linear and the nonlinear trends. Which of the two alternatives to use (linear with more data or nonlinear with subsetted datasets) needs to be addressed in the individual modeling situation. For TOC, the change in trend slope estimates over time seems strong, and therefore, a nonlinear approach would probably be the appropriate choice.

### Including other explanatory variables

In the present study, we did not include any other explanatory variable except time. GWR models are equipped to include additional explanatory variables and can also be fitted with geographical windows of different sizes (so-called mixed GWR; Fotheringham et al., 2002). This would provide the possibility to adjust or normalize data with the aid of local conditions for individual lakes, e.g., local land use or levels of important background variables such as water temperature. How well this works when data are sparse needs to be evaluated in a separate study.

### Large-scale drivers of brownification

While we did not try to make a comprehensive analysis of trends in brownification in Swedish lakes, the modeling results obtained using TOC as a proxy revealed clear large-scale patterns in the prevailing trends. In particular, downward trends emerged in the alpine region and in south-east Sweden, while in the south-west and north-east, decreasing trends were observed during the first years of the study period, but in the last decade, positive changes in TOC were predominant. Although the trends in TOC were generally nonlinear, the GWR analyses managed to identify a large-scale geographical pattern of areas of increasing versus decreasing trends. In the next stage of the analysis, this geographical pattern can be related to levels or trends of land-use activities, catchment characteristics, climatic variables, and acid deposition patterns.

## Conclusions

Data from monitoring programs with low temporal frequency, but covering many sites, are often not considered in trend evaluation. Here, we show that such programs can carry unique and important information about regionally differentiated trends, which will be valuable guidance to distinguish between potential causes of the trend and allow better management decisions.

## Supplementary Information

Below is the link to the electronic supplementary material.

Supplementary file1 (DOCX 1277 KB)

## Data Availability

The data and all used R code that supports the findings of this study are available online (von Brömssen et al., 2023).
