# Supplementary material for: Temporal trend evaluation in monitoring programs with high spatial resolution and low temporal resolution using geographically weighted regression models
Source: Environ Monit Assess. 2023 Apr 10;195(5):547. doi: 10.1007/s10661-023-11172-2 (PMC10083161; doi:10.1007/s10661-023-11172-2)
Supplement: Supplementary file 1 — Supplementary file1 (DOCX 1277 KB) [file 10661_2023_11172_MOESM1_ESM.docx]

Supplementary Information: *Temporal trend evaluation in monitoring programs with high spatial resolution and low temporal resolution using geographically weighted regression models*

Table S1. Lake water monitoring stations with high variation in total organic carbon (TOC) concentrations, as identified by the coefficient of variation. Stations are listed from north to south in Sweden

| ID | Name | Coord E | Coord N | Year | TOC | CV_log_ |
| --- | --- | --- | --- | --- | --- | --- |
| 35119 | - | 655052 | 7570545 | 2010 | 1.4 | 1.85 |
|  |  |  |  | 2016 | 0.25 |  |
| 185502 | - | 526538 | 7359291 | 2008 | 1.5 | 1.12 |
|  |  |  |  | 2013 | 0.7 |  |
|  |  |  |  | 2020 | 0.25 |  |
| 261715 | - | 529431 | 7357459 | 2008 | 1.7 | 1.26 |
|  |  |  |  | 2014 | 0.9 |  |
|  |  |  |  | 2020 | 0.25 |  |
| 217865 | - | 518235 | 7348928 | 2009 | 6 | 1.05 |
|  |  |  |  | 2015 | 1.4 |  |
|  |  |  |  | 2021 | 1.3 |  |
| 183934 | - | 520715 | 7326316 | 2011 | 0.6 | 3.05 |
|  |  |  |  | 2017 | 5.2 |  |
| 185621 | - | 370414 | 6979324 | 2008 | 1.3 | 1.21 |
|  |  |  |  | 2015 | 0.25 |  |
|  |  |  |  | 2021 | 0.25 |  |
| 261367 | Noren38 mitt | 506368 | 6531504 | 2012 | 4 | 2.03 |
|  |  |  |  | 2019 | 24.4 |  |
| 261288 | Iglasjön | 340364 | 6352380 | 2010 | 1.8 | 2.45 |
|  |  |  |  | 2016 | 0.25 |  |
| 260396 | Greppen | 335426 | 6344076 | 2010 | 8.8 | 1.1 |
|  |  |  |  | 2016 | 2.5 |  |
| 261296 | Vrångsgöl | 543739 | 6320860 | 2009 | 33.7 | 1.29 |
|  |  |  |  | 2015 | 137 |  |
| 260621 | Lilla Gäddsjön | 509151 | 6254606 | 2010 | 108 | 1.32 |
|  |  |  |  | 2016 | 26.1 |  |


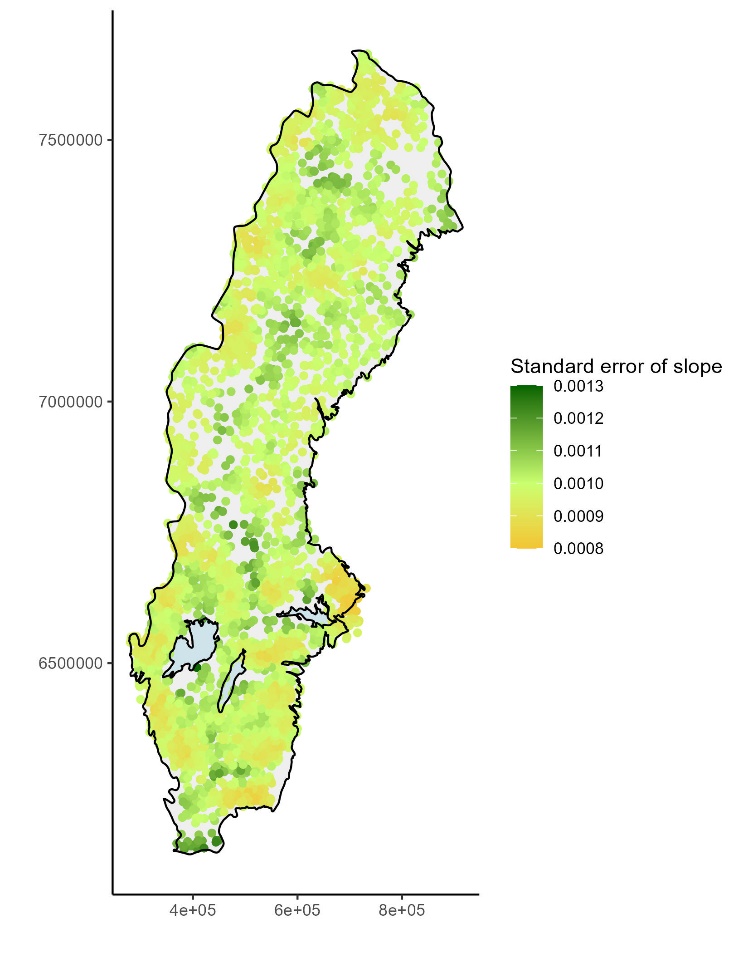


Figure S1. Estimated standard errors of the trend slopes of log-transformed and mean-centered total organic carbon (TOC) concentrations from 2008 to 2021 in geographically weighted regression analysis based on mean-centered data using a cross-validated k-nearest-neighborhood (knn) of 370 observations.


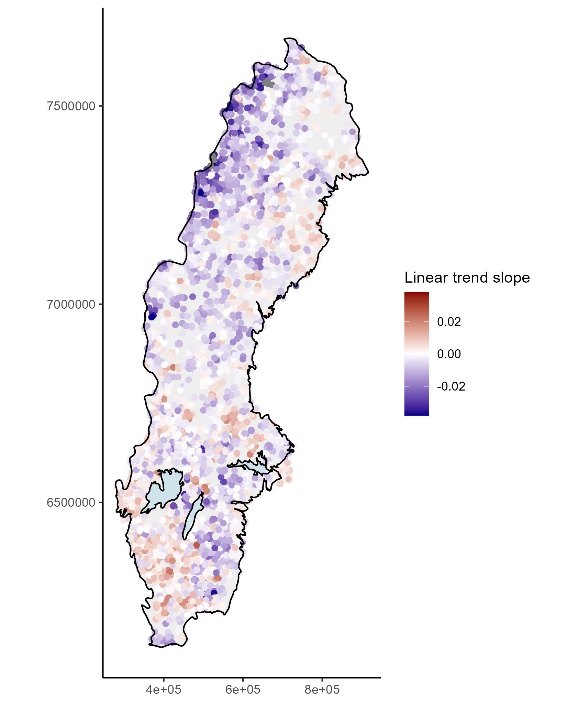

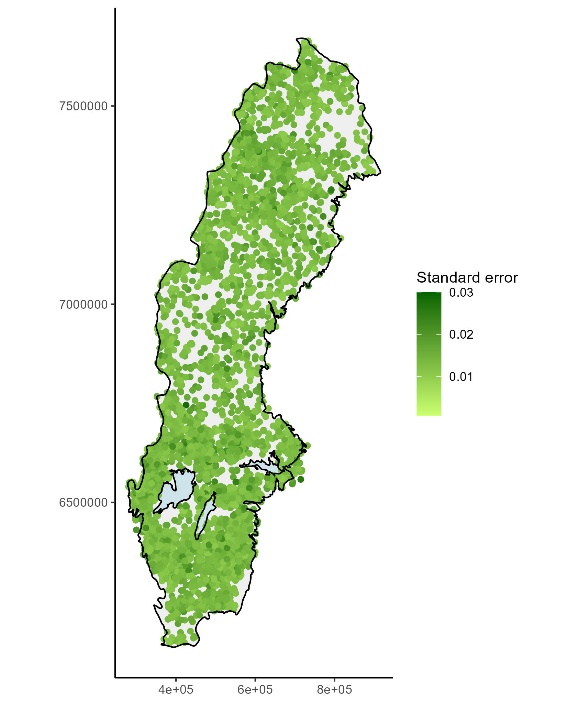


Figure S2. (left) Estimated linear trend slopes of log-transformed total organic carbon (TOC) concentrations from 2008 to 2021 in geographically weighted regression analysis based on data that is not station-wise mean-centered using a cross-validated k-nearest-neighborhood (knn) of 23 observations and (right) the standard error of the same model.


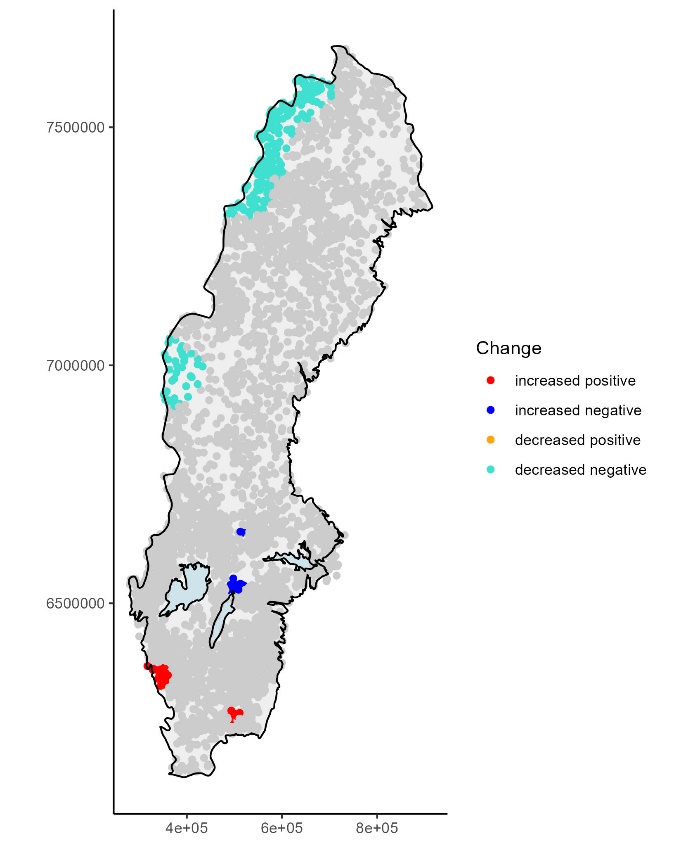

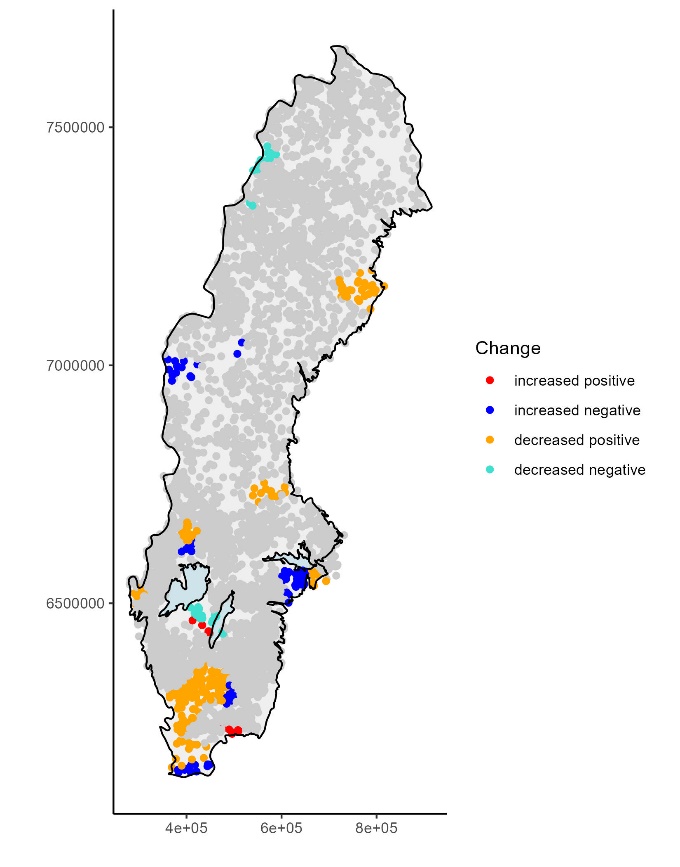


Figure S3. (Left) Changes in slope coefficient estimates when stations with CV_log-normal_ higher than 0.8 were removed and (right) when STL data were added to the SLS data. “Increased positive/negative” indicates that the trend was positive/negative using SLS data alone and was reinforced by STL data. “Decreased positive/negative” indicates that the trend was positive when using SLS data and inclusion of STL data weakened that trend. Only differences between the SLS slope estimate and the combined slope estimate larger than 0.001 units are presented.


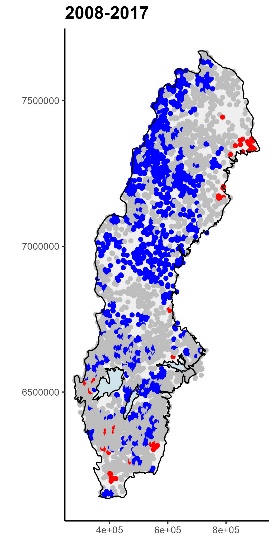

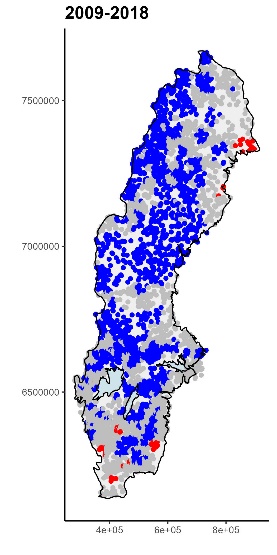

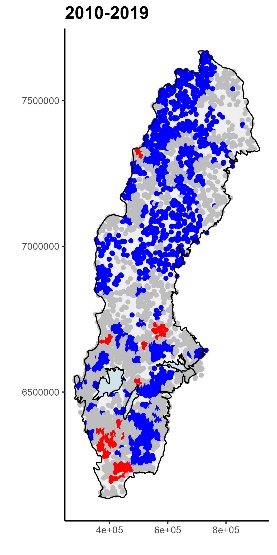

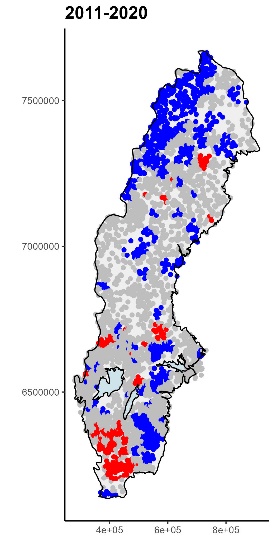

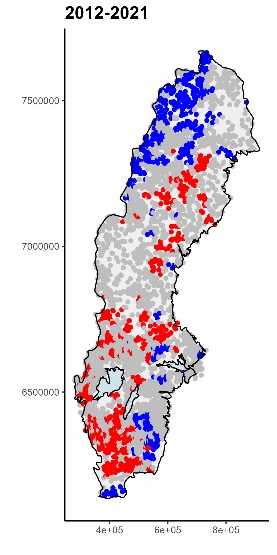


Figure S4. Distribution of slopes significant at 5% level in geographically weighted regression analysis in five 10-year temporal windows, where blue indicates significant downward trend, red significant upward trend, and gray no significant trend.


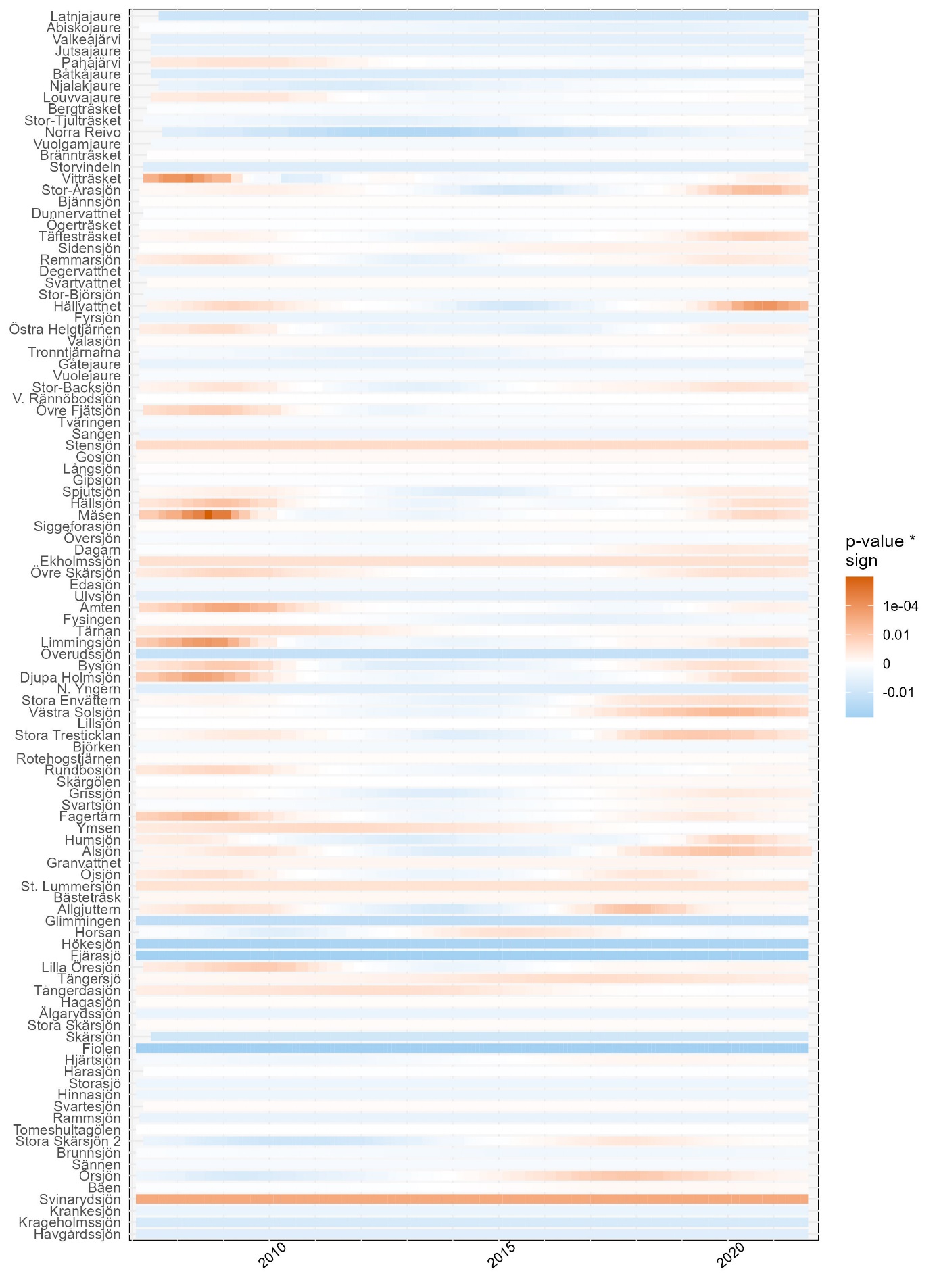


Figure S5. Nonlinear trends in TOC concentration in lakes in the STL program, where blue indicates decreases, red increases, and white no change. The darker the color the more significant the change.
